# Novel *CIC* Point Mutations and an Exon-Spanning, Homozygous Deletion Identified in Oligodendroglial Tumors by a Comprehensive Genomic Approach Including Transcriptome Sequencing

**DOI:** 10.1371/journal.pone.0076623

**Published:** 2013-09-27

**Authors:** Sophie Eisenreich, Khalil Abou-El-Ardat, Karol Szafranski, Jaime A. Campos Valenzuela, Andreas Rump, Janice M. Nigro, Rolf Bjerkvig, Eva-Maria Gerlach, Karl Hackmann, Evelin Schröck, Dietmar Krex, Lars Kaderali, Gabriele Schackert, Matthias Platzer, Barbara Klink

**Affiliations:** 1 Institut für Klinische Genetik, Medizinische Fakultät Carl Gustav Carus, Technische Universität Dresden, Dresden, Germany; 2 Leibniz Institute for Age Research, Fritz Lipmann Institute (FLI), Jena, Germany; 3 Institut für Medizinische Informatik und Biometrie, Medizinische Fakultät Carl Gustav Carus, Technische Universität Dresden, Dresden, Germany; 4 Department of Biomedicine, University of Bergen, Bergen, Norway; 5 Klinik und Poliklinik für Neurochirurgie, Universitätsklinikum Carl Gustav Carus, Technische Universität Dresden, Dresden, Germany; University of Navarra, Spain

## Abstract

Oligodendroglial tumors form a distinct subgroup of gliomas, characterized by a better response to treatment and prolonged overall survival. Most oligodendrogliomas and also some oligoastrocytomas are characterized by a unique and typical unbalanced translocation, der(1,19), resulting in a 1p/19q co-deletion. Candidate tumor suppressor genes targeted by these losses, *CIC* on 19q13.2 and *FUBP1* on 1p31.1, were only recently discovered. We analyzed 17 oligodendrogliomas and oligoastrocytomas by applying a comprehensive approach consisting of RNA expression analysis, DNA sequencing of *CIC*, *FUBP1*, *IDH1/2*, and array CGH. We confirmed three different genetic subtypes in our samples: i) the “oligodendroglial” subtype with 1p/19q co-deletion in twelve out of 17 tumors; ii) the “astrocytic” subtype in three tumors; iii) the “other” subtype in two tumors. All twelve tumors with the 1p/19q co-deletion carried the most common *IDH1* R132H mutation. In seven of these tumors, we found protein-disrupting point mutations in the remaining allele of *CIC*, four of which are novel. One of these tumors also had a deleterious mutation in *FUBP1*. Only by integrating RNA expression and array CGH data, were we able to discover an exon-spanning homozygous microdeletion within the remaining allele of *CIC* in an additional tumor with 1p/19q co-deletion. Therefore we propose that the mutation rate might be underestimated when looking at sequence variants alone. In conclusion, the high frequency and the spectrum of *CIC* mutations in our 1p/19q-codeleted tumor cohort support the hypothesis that *CIC* acts as a tumor suppressor in these tumors, whereas *FUBP1* might play only a minor role.

## Introduction

Gliomas are the most common primary brain tumors in adults. The World Health Organization (WHO) classification divides gliomas into three main subgroups: astrocytomas, oligodendrogliomas, and oligoastrocytomas (mixed gliomas). It further distinguishes between four malignancy grades (WHO grades I–IV). Gliomas exhibiting oligodendroglial features include oligodendrogliomas (WHO grade II) and anaplastic oligodendrogliomas (WHO grade III) as well as oligoastrocytomas (WHO grade II), anaplastic oligoastrocytomas (WHO grade III) and glioblastomas with an oligodendroglial component (GBMO, WHO grade IV) [1]. Oligodendroglial tumors account for 15-20% of all gliomas [2,3].

The identification of the genes targeted by complete 1p/19q co-deletion, a characteristic of oligodendrogliomas, has been a long-standing quest. Combined loss of whole chromosome arms 1p and 19q is the most frequently detected genetic imbalance in oligodendroglial tumors, occurring in 60-90% of oligodendrogliomas and 30-50% of oligoastrocytomas while they are rarely found in GBMO [4-6]. The 1p/19q co-deletion is due to an unbalanced translocation, der(1;19)(q10;p10) [7,8] and has been highly associated with chemosensitivity and a less aggressive course of progression [3,9-11]. Thus, the co-deletion has become an important prognostic and predictive marker.

Recurrent mutations in the capicua transcriptional repressor gene (*CIC*), on 19q and to a much lesser extent in the far upstream element (FUSE) binding protein 1 gene (*FUBP1*) on 1p31.1 have only recently been identified in oligodendrogliomas by using DNA next generation sequencing [12]. Based on the assumption that the 1p/19q co-deletion might help to unmask mutations that result in tumorigenesis, numerous efforts were made in the past to identify putative tumor-associated genes, but with limited success. Therefore, the detection of mutations in *CIC* and *FUBP1* marks an important step in deciphering the process of oligodendroglial tumor development.

Genomic sequencing has also led to the identification of mutations of the isocitrate dehydrogenase genes (*IDH1/2*) in human gliomas. These mutations preferentially occur in lower grade gliomas, including 80-92% of oligodendrogliomas [5,13-15]. Most 1p/19q co-deleted tumors analyzed so far display *IDH1/2* mutations [14,16]. However, *IDH1/2* mutations are not exclusively found in oligodendroglial and oligoastrocytic gliomas, but also in the majority of grade II and III astrocytic tumors, indicating the existence of a common initiating event among these histologically and clinically diverse glioma subtypes [6].

In an effort to further characterize oligodendroglial tumors, we analyzed a set of 17 oligodendrogliomas and oligoastrocytomas by applying a comprehensive approach of genome-wide profiling by array comparative genomic hybridization (array CGH), expression analyses by transcriptome next generation sequencing (RNA-seq) and DNA Sanger sequencing for mutations in *CIC*, *FUBP1* and *IDH1/2.*

## Materials and Methods

### Ethics statement

Tissue and blood samples were obtained from 17 patients undergoing surgery for oligodendroglial tumor removal at the Klinik und Poliklinik für Neurochirurgie, Universitätsklinikum Carl Gustav Carus (Dresden, Germany). Patients had given their prior informed written consent for use of the material for research purposes. Procedures were approved for this project by the Regional Ethics Committee Dresden, Germany (EK 179082004).

### Tumor samples and clinico-pathological grouping

The tumor material was inspected and morphologically classified by neuropathologists. All tumor samples were re-evaluated by experts in the German Brain Tumor Reference Center. Demographic, diagnostic and follow-up information was retrieved from medical records. Seventeen samples of oligodendroglial tumors were analyzed including nine oligodendrogliomas and eight oligoastrocytomas. Tumor cell content was histologically determined in each sample and amounted to at least 80%. Clinical data are summarized in Table S1. The mean age of the total cohort was 43.5 years, which corresponded to previously published studies [13,17]. The cohort was composed of 7 males (41%) and 10 females (59%). There were no differences in age (47.0 years compared to 39.6 years, U-Test, p=0.11) and sex distribution (Fisher’s exact Test, p=1.0) between patients with oligodendrogliomas and oligoastrocytomas.

### DNA and RNA extraction

DNA was extracted from freshly frozen tumor material by phenol-chloroform using standard procedures and from blood using the QIAamp DNA Blood Mini Kit (QIAGEN GmbH, Hilden, Germany) according to the manufacturer’s protocol. RNA from freshly frozen tumor tissue was extracted with the QIAGEN miRNeasy mini Kit according to the manufacturer’s protocol. RNA quality was evaluated using an Agilent RNA 6000 Nano chip on a 2100 Bioanalyzer and RNAs with an RNA integrity number (RIN) below 7.5 were excluded.

### Molecular Karyotyping using array CGH

Array CGH of 17 tumor samples was performed on Agilent’s SurePrint G3 Human CGH Microarray Kit 2x400K (Design ID021850, Agilent, Santa Clara, CA, USA) according to the manufacturer’s protocol with the exception that the labeling of reference and test DNAs was inverted. Scanning of the hybridized arrays was carried out on an Agilent microarray scanner. Raw data were processed by the Feature Extraction 9.5 (Agilent) software and normalization was performed using the default settings. Agilent’s Genomic Workbench Standard Edition 5 0.14 was used in order to determine deleted and amplified regions based on the draft reference human genome version NCBI36/hg18. For the detection of copy number variations (CNVs) the ADM-2 algorithm was applied. A minimum of 5 consecutive probes had to be affected and the threshold for aberration detection was set to 5.9. All chromosomes were in addition visually checked for aberrations in the chromosome view tab.

### Mutation analysis

The following targets were PCR-amplified from tumor DNA samples (n = 17) and tested for mutations by Sanger sequencing: *CIC* (all 20 exons), *FUBP1* (all 20 exons), *IDH1* (codon R132) and *IDH2* (codon R172). Primers used are listed in Table S2. Mutations identified in these targets were confirmed by sequencing a second, independent PCR-product from the same tumor DNA. The somatic status of the confirmed mutations was verified by Sanger-sequencing of the corresponding regions in genomic DNA from matching blood samples. Functional effects of amino acid substitutions were predicted by using PolyPhen-2 version 2.2.2 (http://genetics.bwh.harvard.edu/pph2/), Mutation Taster (http://www.mutationtaster.org), and Mutation *Assessor* (http://mutationassessor.org) [18-20]. In cases where the verdict differed between the three algorithms, we considered the results of the two in agreement.

### Expression analysis

Expression analysis was carried out on the tumor samples for which RNA of sufficient quantity and quality was available (n = 13). Additionally we analyzed RNA of three commercial normal brain controls. Transcriptome next generation sequencing (RNA-seq) was performed using a 100nt approach on the Illumina HiSeq 2000 platform. RNA-seq libraries were prepared using RNA Sample Prep kit v1 (Illumina, San Diego, USA) and sequenced 100 nt, using TruSeq SBS kit v3-HS, to reach a depth of at least 25 million read pairs per sample. We mapped reads to the annotated human transcripts (NCBI RefSeq transcripts, obtained via UCSC repository, 20120228) using the SOAP software (2.21 release; http://soap.genomics.org.cn) with default parameters, multi-threaded, and discarded ambiguous mappings. For evaluation, the expression counts were normalized to RPKM = Reads Per Kilobase of exon model per Million mapped reads (gene counts/total counts of each sample) as described [21]. Additionally RNA was analyzed using SurePrint G3 Human Gene Expression 8x60K Microarrays, Design ID028004 (Agilent, Santa Clara CA, USA) according to the manufacturer’s protocol and data was analyzed using R package *limma* version 3.10.3.

### Validation of an exon-spanning deletion in *CIC*

An exon-spanning deletion in one tumor (BT1) was validated using quantitative RealTime-PCR for several exons of the *CIC* gene and regions situated 3’ of *CIC* using MESA GREEN qPCR MasterMix plus for SYBR® Assay No ROX (Eurogentec, Seraing, Belgium) according to the manufacturer’s instructions. Primers used are listed in Table S2.

### Statistical analysis

Fisher’s exact test was applied to analyze the association between the different aberrations. Mann-Whitney-U test and Fisher’s exact test were carried out on the age and sex distributions among the tumor groups, respectively. For expression analyses of RNA-seq data, the log-scaled expression counts, which showed Gaussian-shape variation across samples of the same class, were used in Student t-Test statistics combined with Bonferroni correction. Agilent expression array datasets were normalized between the arrays using the *limma* package for R. Then a linear model was fitted for each gene and for the comparison between tumor groups and normal brain. The resulting p-values were adjusted using the Benjamini and Hochberg correction.

## Results

We analyzed a cohort of 17 oligodendroglial tumor samples, consisting of nine cases diagnosed as oligodendroglioma (O) and eight as oligoastrocytoma (OA) for copy number variations (CNV); *CIC*, *FUBP1* and *IDH1/IDH2* mutations; and gene expression changes. Results are summarized in Table 1.

**Table 1:** Overview of genetic aberrations in oligodendrogliomas and oligoastrocytomas analyzed in this study.

				***CIC* mutation**		***FUBP1* mutation**	
**Case ID**	**WHO classification**	**Genetic subtype**	***IDH1* mutation**	**position**	**evaluation**	**position**	**evaluation**
BT1	O III	oligo	c.395G>A	**del exon 2-20**	**Deletion**	wt	-
BT2	OA II	oligo	c.395G>A	**c.4436A>C; p.(Gln1479Pro)**	**Missense mutation [PolyPhen-2 score: 0.967]**	wt	-
BT3	O III	oligo	c.395G>A	wt	-	wt	-
BT4	O III	oligo	c.395G>A	**c.1135-1G>A**	**Splicesite mutation**	**c.1041G>A; p.(Gln347=)**	**Silent mutation**
BT5	O II	oligo	c.395G>A	c.604C>T; p.(Arg202Trp)	Missense mutation [PolyPhen-2 score: 1.000]	**c.1041+14T>G**	**Intronic mutation**
BT6	OA III	oligo	c.395G>A	wt	-	wt	-
BT7	O III	oligo	c.395G>A	c.3347dupC; p.(Ser1117Lysfs*34)	Frameshift mutation	c.1623C>G; p.(Tyr541*)	**Nonsense mutation**
BT8	OA II	oligo	c.395G>A	wt	-	wt	-
BT9	OA II	oligo	c.395G>A	wt	-	wt	-
BT14	O III	oligo	c.395G>A	c.601C>T; p.(Arg201Trp)	Missense mutation [PolyPhen-2 score: 1.000]	wt	-
BT15	O II	oligo	c.395G>A	c.3131C>A; p.(Ser1044*)	**Nonsense mutation**	wt	-
BT16	O II	oligo	c.395G>A	**c.4452C>A; p.(Phe1484Leu)**	**Missense mutation [PolyPhen-2 score: 0.965]**	wt	-
BT11	OA II	astro	c.395G>A	**c.3793A>G; p.(Met1265Val)**	**Missense mutation [PolyPhen-2 score: 0.000]**	wt	-
BT12	OA III*	astro	wt	wt	-	wt	-
BT17	OA II	astro	c.395G>A	**c.1466G>C; p.(Gly489Ala)**	**Missense mutation [PolyPhen-2 score: 0.037]**	wt	-
BT13	OA II	other	wt	wt	-	wt	-
BT10	O III	other	wt	wt	**-**	wt	**-**

Mutations in bold indicate novel mutations not yet described in the literature. * BT12 was initially classified as OAIII and later re-classified as A III by a second neuropathologist. **bbreviations**A: O II = oligodendroglioma WHO grade II, O III = anaplastic oligodendroglioma WHO grade III, OA II = oligoastrocytoma WHO grade II, OA III = anaplastic oligoastrocytoma WHO grade III, oligo = “oligodendroglial“ genetic subtype characterized by the co-deletion of chromosome arms 1p and 19q, astro = “astrocytic“ subtype with a gain of chromosome 7 or the combined gain of chromosome 7 and loss of chromosome 10, other = “other” subtype characterized by aberrations not typically found in gliomas, wt = wild typ.

By array CGH, we observed the three distinct genetic subtypes that we have previously described for oligodendroglial tumors [4]. The majority of the O (8/9) and half of the OA (4/8) carried the 1p/19q co-deletion (combined loss of the entire chromosome arms 1p and 19q) typically found in O. These cases were therefore genetically classified as the “oligodendroglial” subtype. Three OA, but none of the O displayed aberrations characteristic of astrocytomas (A), such as gain of chromosome 7 or a combined gain of chromosome 7 and loss of chromosome 10. These cases were classified genetically as the “astrocytic” subtype. A single case each of O and OA had none of the aberrations typically found in glial tumors and therefore, these cases were genetically classified as the “other” subtype.

The *IDH1* mutation c.395G>A (R132H) was present in 14 of the 17 oligodendroglial tumors, including all twelve tumors with 1p/19q co-deletion and two tumors of the “astrocytic” genetic subtype. None of the “other” oligodendroglial tumors had mutations in *IDH1*. *IDH2* mutations were not observed in any case.

### Identification of novel point mutations in *CIC*

**Figure 1:**
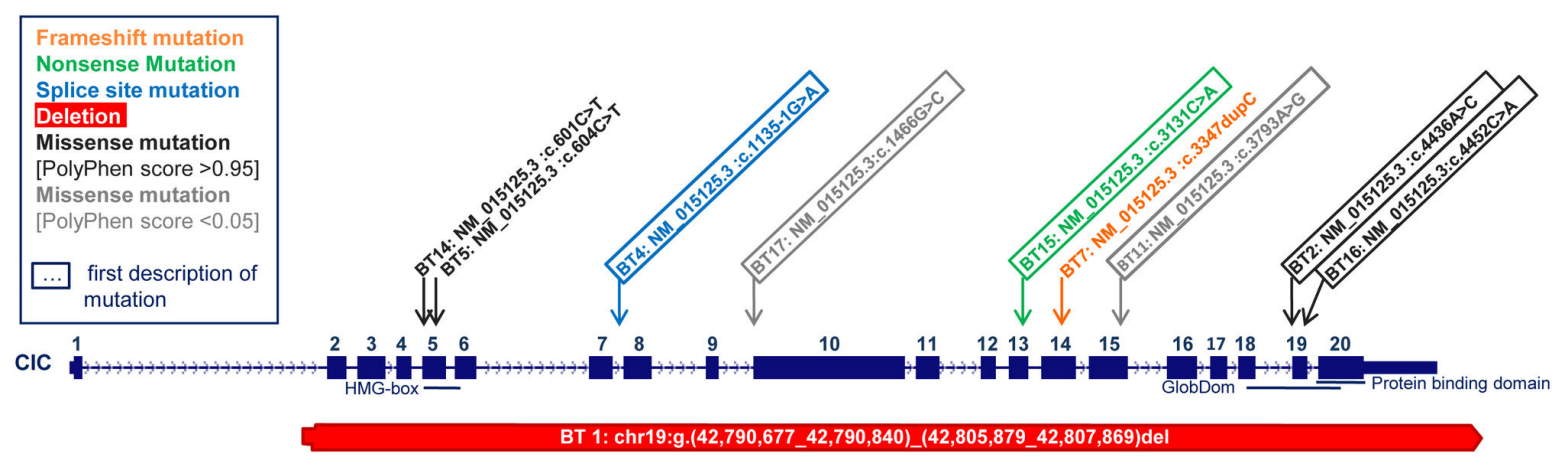
Distribution of *CIC* mutations in this study. The dark blue boxes represent exons. HMG-box denotes the highly conserved DNA-interacting high-mobility group domain. Exon 19 and exon 20 harbor a globular domain (GlobDom). An annotated protein-protein interacting domain is located within exon 20 [22]. Mutations marked by a frame indicate novel mutations not yet described in the literature. While somatic missense mutations were only found in the HMG-box and GlobDom of CIC (BT14,5,2,16), stop-, frameshift- and splice site mutations were found across the CIC protein (BT4,15,7). The two missense mutations that were not located in exons 5 or 19 (BT17,11) were already present in the normal DNAs and had a very low PolyPhen-2 Score, indicating that these SNVs are probably polymorphisms. The red bar represents the exon-spanning deletion in BT1 which extends up to the adjacent *PAFAH1B3* and *PR19* gene (see also Figure 2).

We detected unique somatic single nucleotide variants (SNVs) in *CIC* in 7 of the 12 (58.3%) oligodendroglial tumors with 1p/19q co-deletion. The frequency was 75% (6/8) in cases histologically classified as O, whereas it was only 25% (1/4) in those classified as OA. Four of the seven *CIC* SNVs have not been described so far (Figure 1 and Table 1). The seven SNVs included one frameshift mutation that led to a premature stop codon, one splice-site mutation, one nonsense mutation, and four missense mutations. To evaluate the impact of the four missense mutations on protein structure, we used the PolyPhen-2, Mutation Taster, and Mutation *Assessor* software [18-20]. All four missense mutations were predicted to be “probably damaging” with a PolyPhen-2 score of ≥ 0.96 on a scale of 0 to 1 (Mutation Taster: “disease causing” with a probability >0.99; Mutation *Assessor* : low to medium). Moreover, the missense mutations clustered either in exon 5, the HMG box region, or exon 19, the globular domain (GlobDom) that includes the recently described protein-binding domain of *CIC* [22]. Both gene regions are predicted to be essential to unimpeded protein function (Figure 1). Therefore, all *CIC* mutations found in the 1p/19q-codeleted tumors have the potential to abolish or diminish the function of the remaining allele, either by creating mRNA that is subjected to nonsense mediated decay or by creating a non- or sub-functional CIC protein. None of the SNVs was detected in the DNA from the patients’ blood.

In addition we identified novel *CIC* SNVs in two of the five oligodendroglial tumors lacking the 1p/19q co-deletion (Figure 1). Both SNVs were missense mutations with a very low PolyPhen-2 score (0.037 and 0.000) [Mutation Taster: Disease causing with a probability 0.958 for the former and polymorphism with a probability of 0.999 for the latter; Mutation *Assessor* predicted both SNVs to be neutral], indicating that these SNVs are unlikely to damage protein function, and they were also present in the DNA from the patients’ blood. Moreover, they were located in exons 10 and 15, outside the HMG box and protein binding regions. Missense mutations in these exons have not been described in oligodendroglial tumors so far. Therefore, we assume that these variants are non-functional germ-line variations rather than causally related with tumor origin or growth.

### A novel partial deletion of the remaining *CIC* allele in a 1p/19q co-deleted case

**Figure 2:**
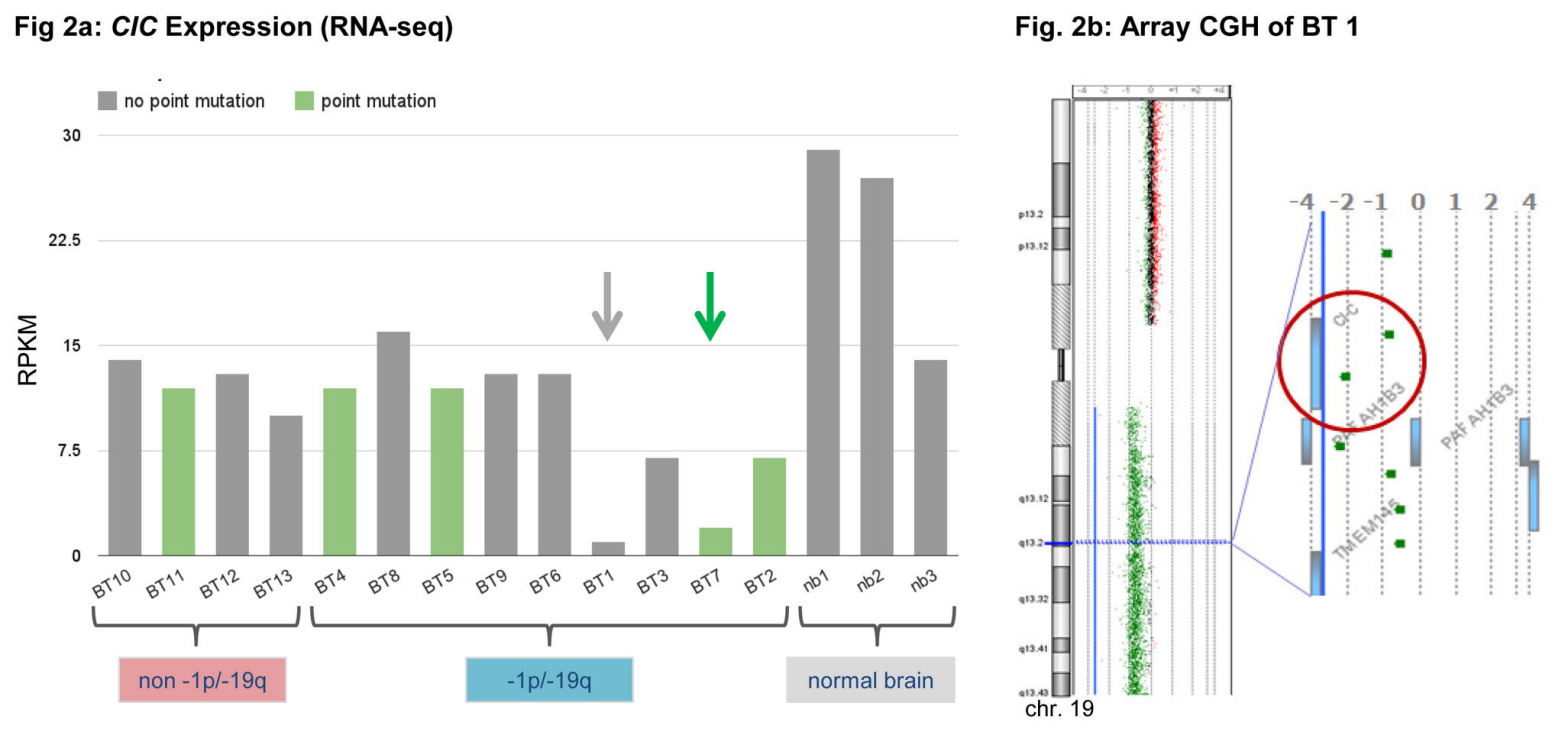
*CIC* Expression analysis and homozygous deletion of *CIC* in array CGH in tumor sample BT1. **2a** Column plot of expression of the *CIC* gene in tumor samples (BT1-15) and normal brain (nb1-3) analyzed using RNA-seq. Tumor samples harboring point mutations are marked as green columns, samples without point mutations as gray columns. Compared to other tumor samples, BT1 (gray arrow) and BT7 (green arrow) show a strong down regulation of *CIC*. This can be explained by a homozygous deletion of *CIC* in BT1 (compare Figure 2b) and nonsense-mediated mRNA decay due to a frameshift mutation in BT7. RPKM = Reads Per Kilobase of exon model per Million mapped reads (gene counts/total counts of each sample). **2b**: Array CGH results of BT1 harboring a partial homozygous deletion of *CIC* (red circle). One oligonucleotide-probe situated within *CIC* and another in *PAFAH1B3* (A_16_P21013642: [hg19] 42,795,949-42,796,008 and A_14_P124269: [hg19] 42804518-42804577) showed a log_2_-ratio of -2, while the whole long arm of chromosome 19 displayed a log_2_-ratio of -0.9 (corresponding to one allele in the tumor and two alleles in the control probe in array CGH). The log_2_-ratio of -2 indicates a homozygous deletion in about 75% of the cell population, corresponding to a background due to normal cells in the tumor tissue. Array CGH narrows the exon-spanning deletion of the second allele of *CIC* to a minimum size of 8.6 kb and a maximum size of 17.3 kb.

**Figure 3:**
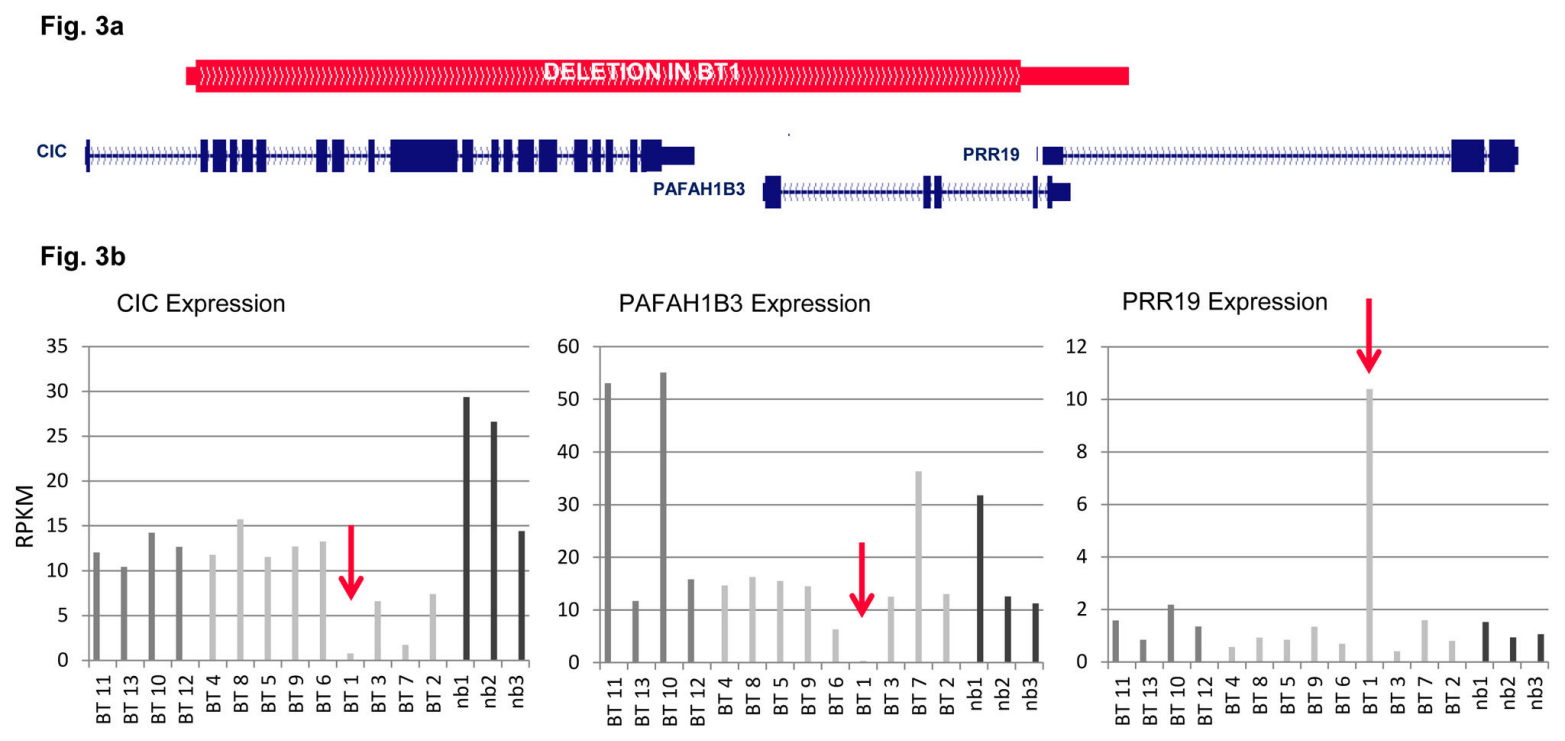
Exon-spanning deletion in BT1 leads to change in expression of *CIC*, *PAFAH1B3* and *PR19*. **3a** Genomic region chr19:42,788,249-42,815,094 that includes the *CIC, PAFAH1B3* and *PR19* gene. The red bar represents the deletion in BT1. The minimum extent (thick) and the maximum extend (thin) of the deletion according to array CGH and qPCR results are shown. **3b**: Expression data (RNA-seq) for tumor samples (BT1-15) with both alleles of 1p and 19q (middle gray), 1p/19q co-deletion (light gray) and normal brain (nb1-3, dark gray). BT1 harboring the exon-spanning deletion shows a strong down regulation of expression in *CIC* and *PAFAH1B3* and an upregulation in *PR19* (red arrows). RPKM = Reads Per Kilobase of exon model per Million mapped reads (gene counts/total counts of each sample).

RNA-seq identified two tumors, BT1 and BT7, with notably decreased *CIC* mRNA levels (Figure 2a, Table S3). Sanger sequencing revealed a frameshift mutation in BT7 (see above), but no mutation in BT1. Nevertheless, array CGH of BT1 showed one oligonucleotide-probe situated within *CIC* and a second probe within the *PAFAH1B3* gene just downstream of *CIC* displayed a log_2_-ratio of -2 (Figure 2b), in contrast to the entire chromosome arm 19q, which displayed a log_2_-ratio of -0.9 to -1. This result indicated a deletion of at least 8.6 kb and a maximum size of 17.3 kb on the remaining 19q-arm. In order to further evaluate and define the breakpoints of the deletion, we performed qPCR on the DNA of BT1 compared to DNA from BT2 with a single copy of 19q and BT11 with two copies. The resulting Ct values of BT1 clearly distinguished between homozygously deleted regions and regions with one remaining copy, where Ct values corresponded to the Ct values of the other 1p/19q-codeleted tumors. The 5’ position of the breakpoint of the deletion was refined to [hg19] chr19:42,790,756-42,790,839 and the 3’ breakpoint to [hg19] chr19:42,805,880-42,808,040 (Figure 3a). We concluded that exons 2-20 of *CIC* and all of *PAFAH1B3* were deleted. These results also correlated with the expression data that showed a significant down-regulation of both *CIC* and *PAFAH1B3* whereas *PRR19*, a gene downstream of *PAFAH1B3* and presumably outside of the deletion, was upregulated (Figure 3b, Table S3).

### Identification of novel somatic mutations in *FUBP1*

We sequenced each of the 20 coding exons of *FUBP1* in all 17 tumors, including exon 6, which is flanked by repetitive poly-A sequences. Three of the 17 tumors displayed somatic SNVs in *FUBP1* that were not yet described in the literature. However, only one of these SNVs, a nonsense mutation in exon 17 (BT7), was definitively protein truncating. The other two SNVs comprised a silent mutation in exon 12 (BT4) and an intronic mutation fourteen bases downstream of exon 12 (BT5). Neither SNV was present in the DNA obtained from the patients’ blood; their relevance remains unclear. All three of these tumors were histologically diagnosed as oligodendrogliomas and harbored the 1p/19q co-deletion and mutations in *CIC* (Table 1). No tumors of the “astrocytic” or the “other” subtype exhibited mutations in the *FUBP1* gene.

### Validation of *CIC* and *FUBP1* mutations in the TCGA cohort

To check the prevalence of the mutations reported here in a larger cohort, we queried the Cancer Genome Atlas (TCGA) dataset of 213 low grade gliomas with complete information through the cBioPortal for Cancer Genomics of the Memorial Sloan-Kettering Cancer Center (http://www.cbioportal.org) [23] (see Supplement information S1). Sixty-four of 213 gliomas had the 1p/19q co-deletion and of those, 33 carried a mutation in *CIC* (see Table S4). Among the *CIC* mutations, we found one case carrying the amino acid substitution R202W, which corresponds to the mutation found in tumor BT5 in our set. The rest of the mutations reported here were not found in the TCGA dataset. There was no report of a microdeletion in *CIC*. However, we identified two cases with 1p/19q co-deletion and without mutation in *CIC* that had marked down-regulation (more than 2.5-fold) of mRNA levels. In the case of *FUBP1*, 20 cases with a 1p/19q co-deletion carried mutations in this gene, none of which were reported in our cohort. Among the 20 cases with *FUBP1* mutations, 14 also carried mutations in *CIC*.

### Expression of *CIC* and *FUBP1* in oligodendroglial tumors relative to 1p/19q status

There were no significant differences in *CIC* expression when comparing 1p/19q co-deleted tumors with tumors without co-deletion (RNA-seq: fold-difference between groups = 0.73, p = 0.14; Agilent 8x60 k: fold-change = 0.52, p = 0.2) (Figure 2). For *FUBP1*, RNA-seq and Agilent 8x60 k data indicated a significantly reduced expression in 1p/19q co-deleted tumors compared to tumors without co-deletion (RNA-seq: fold-change = 0.38, p = 0.004; Agilent 8x60 k: fold-change = 0.38, p = 0.018). However, there was no difference in expression of *FUBP1* between 1p/19q co-deleted tumors and normal brain controls (RNA-seq: fold-change = 0.96, p = 0.35, Agilent 8x60 k: fold-change = 0.54, p = 0.175).

### Association between histology, 1p/19q co-deletion and somatic mutations

All twelve tumors with 1p/19q co-deletion (8 O and 4 OA) also harbored the *IDH1* R132H (c.395G>A) mutation (Fisher’s exact test, p = 0.0147). We found somatic protein altering *CIC* mutations in 8 of 12 1p/19q-codeleted tumors (7 point mutations and one microdeletion), but in none of the five tumors of the “astrocytic” and “other” genetic subtypes (Fisher’s exact test, p = 0.0294). There was no association between somatic *CIC* and *IDH1* mutation (Fisher’s exact test, p=0.206). The proportion of somatic *CIC* mutations was higher in O (7/9, 78%) as compared to OA (1/8, 12.5%) (Fisher’s exact test, p = 0.015), even when considering only tumors with 1p/19q co-deletion (7/8 versus 1/4, Fisher’s exact test, p = 0.067). *FUBP1* mutations only occurred in tumors harboring a 1p/19q co-deletion and a *CIC* mutation (Fisher’s exact test, p = 0.082). Accordingly, there was a concurrence in the incidence of CIC and FUBP1 mutations in the TCGA cohort of low grade gliomas (odds-ratio 3.79; Fisher exact test p-value = 0.0006). The presence of *CIC* and/or *FUBP1* mutations was not associated with tumor malignancy grade (Fisher’s exact test, p = 1 and p = 0.576). Because of the small sample size and the fact that all except for two patients in our cohort are still alive, we did not test for associations between genetic changes and survival. However, the only patient with a tumor harboring protein altering mutations in both *CIC* and *FUBP1* died only three months after surgery. 

## Discussion

Recently, *CIC* and *FUBP1* have been identified as candidate tumor suppressor genes in oligodendrogliomas with the typical 1p/19q co-deletion [12]. In an attempt to uncover the extent and diversity of these mutations, and their impact on gene expression, we carried out a targeted analysis of 17 oligodendroglial tumors. The assessment of mutations in *CIC* and *FUBP1* using a comprehensive approach combining high-resolution array CGH, sequencing and expression data was the main objective of this study.

Through array CGH, three distinct genetic subtypes that we had previously reported could be confirmed in the 17 oligodendroglial tumors [4]. (i) Tumors carrying the 1p/19q co-deletion typically found in O were assigned to the “oligodendroglial” subtype. In our tumor set, 8 out of 9 O and 4 out of 8 OA carried this co-deletion compared to 60-90% of O and 30-50% of OA reported in the literature [5,6]. (ii) Three OA, but no O showed aberrations characteristic of astrocytomas (for example gain of chromosome 7 or a combined gain of chromosome 7 and loss of chromosome 10) and were genetically classified as the “astrocytic” subtype (iii). One O and one OA showed none of these aberrations and were therefore classified as the “other” subtype. We identified the *IDH1* c.395G>A mutation in 14 of the 17 oligodendroglial tumors (82.4% in total, 8/9 O, 6/8 OA), including all 12 tumors with the 1p/19q co-deletion (100%) and two of the three tumors with the “astrocytic” genetic subtype (66.7%), while none of the tumors of the “other” subtype harbored this mutation. This is in accordance with recent findings, where *IDH* mutations were identified in about 75-94% of oligodendrogliomas and 71-98% of oligoastrocytomas [5,13-15]. A strong correlation between the 1p/19q co-deletion and mutation of *IDH1* was previously reported [24], and is consistent with our findings (Fisher’s exact test, p=0.015).

We detected protein altering somatic mutations in the remaining *CIC* allele in eight of the 1p/19q-codeleted tumors, including five novel mutations, and the first description of an exon-spanning deletion. The frequency of mutations in *CIC* in our study corroborates previously published data [12,13,17,22]. In contrast to Yip et al. [22], we did not observe an association between somatic *CIC* and *IDH1* mutation (p=0.206), but were able to confirm that somatic *CIC* mutations were associated with the 1p/19q co-deletion (p=0.0294). Since there is a strong association between 1p/19q co-deletion and *IDH1* mutation, the association of *CIC* mutations with *IDH1* mutation reported by Yip et al. is more likely due to the association with the 1p/19q co-deletion. In agreement with previous studies, we also found a higher occurrence of *CIC* mutations in O (78%) as compared to OA (12.5%) [12,13,17,22]. However, this finding was not significant if only 1p/19q co-deleted tumors were considered (Fisher’s exact test, p = 0.067), and may only reflect the higher occurrence of 1p/19q co-deletion in O compared to OA.

According to the two-hit hypothesis, tumor initiation generally requires both copies of a tumor suppressor gene to be inactivated [25]. In oncogenesis, the first hit could be acquired through the deletion of one allele via a gross chromosomal event, such as loss of an entire chromosome or chromosome arm [26,27]. It can be assumed that the translocation between chromosomes 1 and 19 leading to a subsequent loss of 1p and 19q corresponds to this process in oligodendrogliomas. Inactivation of the second allele may arise from mutations resulting in truncation, missense mutations at residues essential for protein function, from deletions or insertions, or from epigenetic silencing [27]. This scenario is exactly the case for the *CIC* mutations detected in our tumor set. All of the somatic *CIC* alterations we found in the remaining allele of 1p/19q-codeleted tumors either led to the truncation of the CIC protein (4/8) or were missense mutations predicted to be damaging by PolyPhen-2 (4/8). We have found a partial deletion of the *CIC* gene - an event not yet reported in the scientific literature - that extends from exon 2 of *CIC* to the adjacent *PAFAH1B3* gene. We evaluated previously published data and confirmed that all reported tumors with 1p/19q co-deletion and *CIC* mutation showed a protein damaging mutation of the remaining allele: all were either truncating mutations (nonsense, frameshift, and splice acceptor mutations), missense mutations that are predicted to be protein damaging (PolyPhen-2 score of at least 99%, disease causing according to Mutation Taster with probability > 0.68; functional impact according to Mutation *Assessor* ranging from low to high), or in-frame-deletions in exons 5 and 20 (see Table S3) [12,13,17,22]. It can therefore be stated that *CIC* mutations are not only frequent in oligodendroglial tumors, but also bear a high potential of inactivating their encoded protein. Our findings together with published data therefore support the hypothesis that *CIC* functions as a tumor suppressor in 1p/19-codeleted oligodendrogliomas and oligoastrocytomas and is commonly altered in these tumors.

All four somatic *CIC* missense mutations in our 1p/19q-codeleted tumors were located in exons 5 and 19. Mutations have been previously shown to cluster in exons 5 and 20 [22]. Exon 5 and parts of exon 6 encode the HMG box domain, which seems to include several mutational hotspots, such as the c.6014C>T (p.Arg201Trp) [12,13,17,22]. Base exchange is very likely to occur at the cytosine at this position, because it is part of a CpG dinucleotide and almost certainly methylated in the mammalian genome. One third of all human point mutations appear in such sites through deamination of the 5-methylcytosine to uracil leading to a transition from CpG to TpG [28]. Yip et al. also described missense mutations in exon 20, where they predicted the location of a protein binding domain [22]. In our cohort, we found missense mutations in exon 19 but not in exon 20. Bettegowda et al. and Jiao et al. found also four missense mutations in exon 19. We therefore assume an association with an annotated GlobDom that includes both exon 19 and 20 (compare Figure 1). Data mining of the existing literature and the TCGA dataset revealed that out of a total of 77 missense mutations with high Polyphen-2 score, 60 were located within the HMG-box (77.9%), 15 within the GlobDom (19.5%) and only two outside of these structural units (compare Table S2) [12,13,17,22].

It is important to point out that we and others have found protein truncating *CIC* mutations (nonsense, frameshift, and splice site mutations) that were located outside exons 5, 19, and 20 (see Table S4). Since a loss of function mutation (which one would expect for tumor suppressor gene to promote cancer) can be acquired through different types of mutations and along almost the whole gene, one would not expect to find mutations (except for missense mutations) only in certain domains of a gene. Our results emphasize that sequencing only “hotspot” regions of the *CIC* gene, such as exons 5 and 20, as indicated by others, may not be sufficient to detect the whole spectrum of *CIC* mutations, some of which are deleterious to the CIC protein function [22]. The results of the low grade brain tumor characterization by TCGA seem to underscore our findings; the mutations in *CIC* in the 213 analyzed samples with complete information are not restricted to exons 5 and 20. The five novel mutations reported here were not found in the TCGA samples including any microdeletions in *CIC*. The lack of microdeletions could be due to the fact that small (intragenic) deletions in *CIC* could be below the resolution limit of the platform used to detect CNVs in the TCGA samples (Affymetrix’s Genome-Wide Human SNP Array 6.0). We have, however, identified two cases within the TCGA dataset with 1p/19q co-deletion, no *CIC* mutations, and very low expression level of *CIC*, which is very similar to our findings in one case with partial deletion of *CIC*. This might indicate a second hit in the *CIC* gene in these tumors – such as a microdeletion below the detection limit - that was missed by the applied methods.

Our tumor set also included two tumor samples that harbor novel SNVs of *CIC* but did not carry the 1p/19q co-deletion. Both SNVs were transmitted via or occurred in the germline as they are present in the DNA from the patients’ blood. They were located in exons 10 and 15, and were missense mutations not likely to be protein damaging according to *in silico* prediction. We therefore assume that these SNVs are rare variants and not mutations in the sense of a pathogenic effect. To the best of our knowledge, only two tumors with a *CIC* mutation that retained both copies of 19q have been described in the literature so far. One reported by Sahm et al. had a missense mutation located in exon 3 [17]. We evaluated this mutation using *in silico* prediction, and found it to not have a deleterious effect on protein function (PolyPhen-2 score 0.000; ‘polymorphism’ according to Mutation Taster [probability 0.894]; see Table S4). The other case, reported by Yip et al., was diagnosed as a WHO IV astrocytoma/gliomatosis cerebri [22]. Using PCR, the authors did not find a 1p/19q co-deletion in this tumor. However, since this method is less sensitive if the relative proportion of tumor to stromal cells is too small, as often the case in gliomatosis cerebri, the 1p/19q co-deletion could simply not have been detected. In addition, we reevaluated the functional effect of all other missense mutations in 1p/19q-codeleted tumors reported so far using *in silico* prediction. Only one additional tumor harbored a missense mutation with Polphen-2 score < 0.01 (Mutation Taster: ‘polymorphism’ with probability 0.996; Mutation *Assessor* : ‘neutral’) [22]. This mutation was also located in exon 3 and the tumor had an additional frameshift mutation in the same allele of the *CIC* gene (see Table S4). Therefore, we assume that the two missense mutations with a low PolyPhen-2 score reported by Yip et al. and Sahm et al. are also rather rare non-functional variants [17,22]. Unfortunately, the authors did not analyze normal tissue from the patients; therefore it is not known, whether these variants were also germline. We propose that the aforementioned *CIC* SNVs in our cases and the reported tumors with low PolyPhen-2 scores do not constitute a ‘hit’ in the Knudson model. Nevertheless, we cannot exclude that these rare variants contribute to tumor development.

Expression analysis showed no significant differences in the transcript level of *CIC* in tumors with and without 1p/19q co-deletion. *CIC* expression did not differ significantly between tumors with and without *CIC* mutation, concordant with recently published data [22]. However, there were two exceptions with notably decreased transcripts: BT1 where the remaining allele of *CIC* was partially deleted and BT7 with a frameshift mutation causing a premature translation-termination codon (PTC) in the remaining allele of *CIC*. In eukaryotes, mRNA harboring PTCs is detected and eliminated by nonsense-mediated mRNA decay [29]; this could explain loss of *CIC* expression in BT7. Our results indicate a tight regulation of the *CIC* promoter activity in oligodendroglial tumors, leading generally to normal transcript level of *CIC* even in tumors with 1p/19q co-deletion.

Mutations of *FUBP1* were recently reported in a subset of oligodendrogliomas [12,17]. In our study, somatic mutations of *FUBP1* on chromosome arm 1p were only found in 3 of 17 tumors (17.6%) and only in tumors of the “oligodendroglial” genetic subtype (3/12, 25%). The frequency of *FUBP1* mutations in our cohort corresponds well to the frequency of *FUBP1* mutations reported in the literature, ranging between 11.1% and 22.2% for O, 6.3% and 12.5% for OA [12,17], and 6.3% and 28.6% for 1p/19q-codeleted tumors [17]. Frameshift and nonsense mutations were the predominant types of alterations found in previous studies [17], but only one of the three mutations in *FUBP1* detected in our tumor set is predicted to generate a truncated protein (BT7). The relevance of the other two mutations is unclear: one was a silent mutation (BT4) and the other occurred in an intronic region (BT5).

*FUBP1* mutations occurred only in tumors that also had both *CIC* and *IDH1* mutations combined with 1p/19q co-deletion, consistent with observations of Sahm et al [17]. Interestingly, the one case that presented deleterious alterations in both *CIC* and *FUBP1* (BT7) was the only recurrent tumor in our cohort, and the patient died only 3 months after surgery. We have previously reported on a different anaplastic oligodendroglioma with the same mutational status that was also a recurrent tumor and had engrafted in mice [30]. Based on the relatively low incidence of *FUBP1* mutations, their concurrence with *CIC* mutations, and their occurrence in recurrent tumors, we hypothesize that *FUBP1* mutations might constitute a later event in oligodendroglioma tumorigenesis.

To date, not much is known about the function of the protein capicua homolog that is encoded by the *CIC* gene in humans. It contains an annotated high mobility group (HMG) domain and was classified as a member of a new Sox-related HMG subfamily [31]. This highly conserved DNA-interacting domain allows the CIC protein to bind nucleosomes and thus to regulate chromosome architecture and gene transcription [32]. Since CIC is predominantly expressed in immature granule cells in the CNS, it is assumed that CIC has a role in CNS development [31]. In addition, CIC function has been explored in *Drosophila* where it regulates terminal and dorso-ventral patterning of the embryonic body by repressing genes downstream of multiple receptor tyrosine kinase (RTK) pathways, such as Torso and EGFR, by binding to octameric sequences in the promoter regions of the affected genes. CIC itself is under negative post-transcriptional control by RTK signaling via MAPK-mediated degradation of the CIC protein by phosphorylation [33-35]. *FUBP1* codes for the far upstream binding protein 1 (FUBP1 also named FBP1), which has an important role in cell proliferation and is implicated in multiple types of cancers [36]. FUBP1 stimulates the transcription of the c-Myc proto-oncogene by binding to the single strand DNA of the far upstream element (FUSE) in the c-Myc promotor region [37,38]. By complexing with the FUBP interacting repressor (FIR) it negatively regulates c-Myc expression [38]. A number of recent reports have indicated that independent of the c-myc pathway, FUBP1 acts as an RNA-binding protein to cellular mRNA or viral RNA [39,40] and is involved in the development of the neural system [36,41,42].

We were able to show here that *CIC* mutations are common in oligodendroglial tumors with 1p/19q co-deletion whereas *FUBP1* mutations seem to occur more rarely. We also broadened the spectrum of *CIC* mutations and provided the first report of a partial homozygous deletion of *CIC* that was not detected by standard sequencing or array CGH alone. Our results highlight the importance of a comprehensive approach for the detection of *CIC* mutations. Based on our results, it is likely that previous sequencing studies have underestimated the frequency of *CIC* mutations. Exome sequencing complemented by detection of smaller rearrangements and deletions, for example, by using paired-end sequencing, may illuminate the full mutational spectrum of *CIC* in human oligodendrogliomas. The high frequency of *CIC* mutations in this and previous studies, their co-occurrence with 1p/19q co-deletion, and their inactivating character support the theory of *CIC* being a tumor suppressor gene in the development of human oligodendrogliomas. However, not all tumors with the 1p/19q co-deletion carried an alteration in the second allele of *CIC* (or *FUBP1*), indicating that other mechanisms or genes are involved in oligodendroglioma development. It will be important to delineate the pathway through which *CIC* acts and to analyze *CIC* mutations in a larger cohort of tumors so that conclusions about their impact on prognosis and treatment can be made. Our results in conjunction with other studies indicate that *CIC* mutations play a critical role in oligodendroglioma development and form a molecular feature distinctive of this glioma subtype.

## Supporting Information

Table S1 **Clinical data and histology of patients from whom tumor material was analyzed.**
(PDF)

Table S2 **Primers used in this study.**
(PDF)

Table S3 **individual expression values of *CIC* and *FUBP* as well as the log_2_ ratios (array CGH) for all tumor samples analyzed.**
(XLSX)

Table S4 ***CIC* mutations reported in the literature and in the cohort analyzed in this study.**
(XLSX)

Information S1 **Querying the Cancer Genome Atlas datasets for low grade glioma.**
(PDF)
