# Supplementary material for: Novel CIC Point Mutations and an Exon-Spanning, Homozygous Deletion Identified in Oligodendroglial Tumors by a Comprehensive Genomic Approach Including Transcriptome Sequencing
Source: PLoS One. 2013 Sep 27;8(9):e76623. doi: 10.1371/journal.pone.0076623 (PMC3785522; doi:10.1371/journal.pone.0076623)
Supplement: Table S1 — Clinical data and histology of patients from whom tumor material was analyzed. (PDF) [file pone.0076623.s001.pdf]

Supplementary Table S1: Clinical data and histology of patients from whom tumor material was analyzed

Case ID	WHO classification	Sex	Age at diagnosis in years	Survival in days*
BT1	O III	f	47	2074
BT2	OA II	f	29	3728
BT3	O III	m	63	3209
BT4	O III	f	51	3318
BT5	O II	f	40	2623
BT6	OA III	m	35	1641
BT7	O III	m	55	72†
BT8	OA II	m	41	1729
BT9	OA II	m	40	1306
BT10	O III	f	5	5963
BT11	OA II	f	34	1139
BT12	OA III	f	60	818
BT13	OA II	f	47	1347
BT14	O III	m	45	1576
BT15	O II	f	56	3502†
BT16	O II	m	43	2373
BT17	OA II	f	31	2360

Abbreviations: O II = oligodendroglioma WHO grade II, O III = anaplastic oligodendroglioma WHO grade III, OA II = oligoastrocytoma WHO grade II, OA III = anaplastic oligoastrocytoma WHO grade III, m = male, f = female, *survival in days from time of surgery to last follow up or death, † = patient died
