# Supplementary material for: Novel CIC Point Mutations and an Exon-Spanning, Homozygous Deletion Identified in Oligodendroglial Tumors by a Comprehensive Genomic Approach Including Transcriptome Sequencing
Source: PLoS One. 2013 Sep 27;8(9):e76623. doi: 10.1371/journal.pone.0076623 (PMC3785522; doi:10.1371/journal.pone.0076623)
Supplement: Table S2 — Primers used in this study. (PDF) [file pone.0076623.s002.pdf]

Supplementary Table S2: Primers used in this study

CIC Primer for Exon Sequencing

Primer	Sequence 5'--> 3'	Product Length
CIC_x1_1_F<br>CIC_x1_B_R	ACCATGTATTCGGCCCACA<br>CTGCACCCCTCACCTTTCTA	334 bp
CIC_x2_F<br>CIC_x2_R	CCTCATCTAGGGTGGGGAAG<br>AGCAACAGCTGCCGACTC	344 bp
CIC_x3_F<br>CIC_x3_R	CAACCAGAGCAAAGGTGAGG<br>GACATGATGGAGAGGAAGCTG	431 bp
CIC_x4_F<br>CIC_x4_R	ACCATGATGATGCGTGAGTT<br>CTTCTCCCGCTGCATTAAAC	326 bp
CIC_x5_F<br>CIC_x5_R	CAAGGAACGGGACTCATCTT<br>AGGGCAACCCTTCACTCAC	394 bp
CIC_x45_F<br>CIC_x45_R	CATGATGATGCGTGAGTTCC<br>AAGTGGGCCTCCTTCACCT	597 bp
CIC_x6_F<br>CIC_x6_R	GTAACGCTGTTGCCTCTTGG<br>AAGCAAGGGGGTAGCTGGT	318 bp
CIC_x7_F<br>CIC_x7_R	GAAATTCCAGCCTGCTTCTG<br>CTGCAGGATAGGTGGCAG	406 bp
CIC_x8_F<br>CIC_x8_R	GAACTGACGCAGGTCTAGGG<br>AAGTTCTGCCCACCCTCTG	398 bp
CIC_x9_F<br>CIC_x9_R	GCCGGTTTGGAGCAGAG<br>ACTATGACCCAACTCCCCAC	344 bp
CIC_x10_1_F<br>CIC_x10_1_R	GATGAATTGAGGCCTTCAGC<br>TCTTGCTCCTCCTTGTTTGG	595 bp
CIC_x10_2_F<br>CIC_x10_2_R	ACCAGGCCCCTCAGTCAT<br>GGTGCCCACCAGTAGGTTAG	469 bp
CIC_x10_3_F<br>CIC_x10_3_R	CTGCTGGGCACTGTGGG<br>AAGAGGCAAGAAAGGAAAAGG	536 bp
CIC_x11_F<br>CIC_x11_R	CCATCCTGCAGTCTGTACCC<br>ATCACGGAAGATGGCAAGAC	445 bp
CIC_x1213_F<br>CIC_x1213_R	TCTGTGTTCCTTGCTTTTGC<br>AAGCAGGAGACCAAGTTAGGG	567 bp
CIC_x14_F<br>CIC_x14_R	GTGCAGCCCCTGCTCTC<br>GGGGAGAAAATTGTTTGCTG	503 bp
CIC_x15_F<br>CIC_x15_R	TCAGATCAACCCAGAGCAGA<br>ACGTCAATGGAGAGGCACA	475 bp
CIC_x16_F<br>CIC_x16_R	CGGGCTCAGATCCAACTCT<br>TCGAAGTCCACTTCTGACAGG	457 bp
CIC_x17_F<br>CIC_x17_R	CTTGGTGGAGCGTGTTAGG<br>CAGGTCTGGCCAAACAGAAG	323 bp
CIC_x18_F<br>CIC_x18_R	AAGAGGAAGAACTCCACGGG<br>GGACCCACCCTACCTCTCTC	329 bp

CIC_x19_F<br>CIC_x19_R	TAGAATGCAGTGAGGGCTTG<br>TTGGAGGGAAAGATGTCTGC	366 bp
CIC_x20_F<br>CIC_x20_R	TTCAGGACCATGGCTTCTTC<br>TTACCCGGGAGGAGATAACC	572 bp

CIC Primer Flanking the Deletion in BT1

Primer	Sequence 5'--> 3'	Product Length
CICBP_x_1F<br>CICBP_x_1R	GTGTTCCAGCTCCCCATTCT<br>AAAGTGAGGTTCAGCCATGC	281 bp
CICBP_x_2F<br>CICBP_x_2R	CAAGCCCCATAGAAGCAAGA<br>AATGAGGCTTTCCTGCCATA	292 bp
CICBP_x_3F<br>CICBP_x_3R	TATGGCAGGAAAGCCTCATT<br>GTCTTCATCGGGGACTCCTT	287 bp
CICBP_x_4F<br>CICBP_x_4R	CTTTGCTGTCAGCCACGAAC<br>AGATGAGTGGAGAGGAGAACC	300 bp
CICBP_x_5F<br>CICBP_x_5R	TCTCCTCTCCACTCATCTTGG<br>GTGGGTGGTTTCTCCCTCCT	381 bp
CICBP_x_6F<br>CICBP_x_6R	CAAGACATCACAGGACCCCTA<br>GGCCTTGGCTATGGATGTAG	284 bp
CICBP_x_7F<br>CICBP_x_7R	AATGAGTGGGTCAGCTCCTG<br>TTCCACGATATTCCCCAAAC	256 bp
CICBP_x_8F<br>CICBP_x_8R	TTATGTCTGTGGCTGCTTGG<br>GGCTCTGAGAGGACGGAAAC	283 bp
CICBP_qLR_F<br>CICBP_qLR_R	TCCCAGCCCGTAGAGGTC<br>CCCACCAATCTCCACCAG	127 bp
CICBP_LRn_F<br>CICBP_LR_R	CTAAGTGCGGAGTAACGGTGGTG<br>TCTGCTTGCCCATTCTTCATTATC	19099 bp
CICBP_LRex_F<br>CICBP_LRex_R	GTCAAGGTGTCGGGGTGCTAAG<br>GCTATTCCACGATATTCCCCAAAC	19173 bp

FUBP1 Primer for Exon Sequencing

Primer	Sequence 5'--> 3'	Product Length
FUBP1_x1_F<br>FUBP1_x1_R	GGTCGCGCAAGAATGTAATAG<br>CCGGAAGAACACCTCTTTCC	340 bp
FUBP1_x2_F<br>FUBP1_x2_R	TCTCTTCCGGATTCCTAGTTG<br>CCCTTTTAGGCCAATATACAGC	498 bp
FUBP1_x3_F<br>FUBP1_x3_R	TGGCAGTTTTGGTTCAAATG<br>AAACCAACTAACTTCAAAGATACCC	272 bp
FUBP1_x4_F<br>FUBP1_x4_R	CTCTTCAAGGTGTTGTCTGTGC<br>TTCCCAAGGGGAGTGATATG	303 bp
FUBP1_x567_F<br>FUBP1_x567_R	AAGCCATTGGGAGACATTAGAC<br>AGGACTTTGAGGGAAGGTGG	641 bp
FUBP1_x6_F	TCACACTGAAAATACCATGAGAATG	108 bp

FUBP1_x6_R	AGTAATTGGCAGAGGAGGTGA	
FUBP1_x89_F<br>FUBP1_x89_R	GGCACCTAATCCTGAACTGG<br>CTCTAACACCATTTCCTTGGC	624 bp
FUBP1_x1011_F<br>FUBP1_x1011_R	CAAAACTCACTGCTGCCAAC<br>TTAAAATGTTTTGAGACATGCTTT	593 bp
FUBP1_x12_F<br>FUBP1_x12_R	CAAAATGATGCTGGTGTTCG<br>AAAGCATCATCAAGCAGACTC	334 bp
FUBP1_x13_F<br>FUBP1_x13_R	GATTCAGGTATATGAGAGCTGGG<br>TTCTTTTCTACTAAAATACGGTCAGAG	363 bp
FUBP1_x14_F<br>FUBP1_x14_R	TGCTGACTAGTAATGATACATTTTCC<br>GGCCCATTTAATTGTGACCA	356 bp
FUBP1_x1516_F<br>FUBP1_x1516_R	TCTCATTATTATGGCAGATGAAATAG<br>ACACTGGGGTAGAGGCAGTG	639 bp
FUBP1_x17_F<br>FUBP1_x17_R	ATATTTGCCAGAGAGCTGGG<br>GGTGCTCCGGGTATTCTTC	419 bp
FUBP1_x18_F<br>FUBP1_x18_R	CTCACTGTCACATTGCAGGC<br>CAACTGTCTTGATATGTTTACAAGTCC	300 bp
FUBP1_x19_F<br>FUBP1_x19_R	TCAACAAGGGCAAACACAAG<br>AATTTAAAAGGCAAACACTCCC	372 bp
FUBP1_x20_F<br>FUBP1_x20_R	TTGCAAATCATGCAAGAAGC<br>AAAACAAACCAATTTTCATTTCTACAC	360 bp

IDH1/2 Primer for Exon Sequencing

Primer	Sequence 5'--> 3'	Product Length
IDH1_x1_F<br>IDH1_x1_R	ACCAAATGGCACCATACGA<br>TTCATACCTTGCTTAATGGGTGT	254 bp
IDH2_x1_F<br>IDH2_x1_R	GCTGCAGTGGGACCACTATT<br>TGTGGCCTTGTACTGCAGAG	293 bp
