# Supplementary material for: Novel CIC Point Mutations and an Exon-Spanning, Homozygous Deletion Identified in Oligodendroglial Tumors by a Comprehensive Genomic Approach Including Transcriptome Sequencing
Source: PLoS One. 2013 Sep 27;8(9):e76623. doi: 10.1371/journal.pone.0076623 (PMC3785522; doi:10.1371/journal.pone.0076623)
Supplement: Information S1 — Querying the Cancer Genome Atlas datasets for low grade glioma. (PDF) [file pone.0076623.s005.pdf]

Supplementary Information S4: Querying the Cancer Genome Atlas datasets for low grade glioma

We queried the 213 low grade glioma samples with complete information (expression, CNV, and SNP) in the Cancer Genome Atlas (TCGA) datasets using the cBioPortal for Cancer Genomics from the Memorial Sloan-Kettering Cancer Center (http://www.cbioportal.org; current as of 19/07/2013). 1p/19q status was determined by downloading the copy number data to the Integrative Genome Viewer (IGV; ver. 2.3.12) and only cases with the loss of one whole long arm of chromosome 19 and one whole short arm of chromosome 1 were considered as having the 1p/19q co-deletion. For mutations in *CIC* and *FUBP1*, only those with an allele frequency ≥ 0.2 were considered as true mutations.
